# New algorithms for identifying the flavour of ${\mathrm {B}} ^0$ mesons using pions and protons

**DOI:** 10.1140/epjc/s10052-017-4731-y

**Published:** 2017-04-12

**Authors:** R. Aaij, B. Adeva, M. Adinolfi, Z. Ajaltouni, S. Akar, J. Albrecht, F. Alessio, M. Alexander, S. Ali, G. Alkhazov, P. Alvarez Cartelle, A. A. Alves, S. Amato, S. Amerio, Y. Amhis, L. An, L. Anderlini, G. Andreassi, M. Andreotti, J. E. Andrews, R. B. Appleby, F. Archilli, P. d’Argent, J. Arnau Romeu, A. Artamonov, M. Artuso, E. Aslanides, G. Auriemma, M. Baalouch, I. Babuschkin, S. Bachmann, J. J. Back, A. Badalov, C. Baesso, S. Baker, W. Baldini, R. J. Barlow, C. Barschel, S. Barsuk, W. Barter, M. Baszczyk, V. Batozskaya, B. Batsukh, V. Battista, A. Bay, L. Beaucourt, J. Beddow, F. Bedeschi, I. Bediaga, L. J. Bel, V. Bellee, N. Belloli, K. Belous, I. Belyaev, E. Ben-Haim, G. Bencivenni, S. Benson, J. Benton, A. Berezhnoy, R. Bernet, A. Bertolin, F. Betti, M.-O. Bettler, M. van Beuzekom, Ia. Bezshyiko, S. Bifani, P. Billoir, T. Bird, A. Birnkraut, A. Bitadze, A. Bizzeti, T. Blake, F. Blanc, J. Blouw, S. Blusk, V. Bocci, T. Boettcher, A. Bondar, N. Bondar, W. Bonivento, I. Bordyuzhin, A. Borgheresi, S. Borghi, M. Borisyak, M. Borsato, F. Bossu, M. Boubdir, T. J. V. Bowcock, E. Bowen, C. Bozzi, S. Braun, M. Britsch, T. Britton, J. Brodzicka, E. Buchanan, C. Burr, A. Bursche, J. Buytaert, S. Cadeddu, R. Calabrese, M. Calvi, M. Calvo Gomez, A. Camboni, P. Campana, D. Campora Perez, D. H. Campora Perez, L. Capriotti, A. Carbone, G. Carboni, R. Cardinale, A. Cardini, P. Carniti, L. Carson, K. Carvalho Akiba, G. Casse, L. Cassina, L. Castillo Garcia, M. Cattaneo, Ch. Cauet, G. Cavallero, R. Cenci, M. Charles, Ph. Charpentier, G. Chatzikonstantinidis, M. Chefdeville, S. Chen, S. F. Cheung, V. Chobanova, M. Chrzaszcz, X. Cid Vidal, G. Ciezarek, P. E. L. Clarke, M. Clemencic, H. V. Cliff, J. Closier, V. Coco, J. Cogan, E. Cogneras, V. Cogoni, L. Cojocariu, P. Collins, A. Comerma-Montells, A. Contu, A. Cook, G. Coombs, S. Coquereau, G. Corti, M. Corvo, C. M. Costa Sobral, B. Couturier, G. A. Cowan, D. C. Craik, A. Crocombe, M. Cruz Torres, S. Cunliffe, R. Currie, C. D’Ambrosio, F. Da Cunha Marinho, E. Dall’Occo, J. Dalseno, P. N. Y. David, A. Davis, O. De Aguiar Francisco, K. De Bruyn, S. De Capua, M. De Cian, J. M. De Miranda, L. De Paula, M. De Serio, P. De Simone, C. T. Dean, D. Decamp, M. Deckenhoff, L. Del Buono, M. Demmer, A. Dendek, D. Derkach, O. Deschamps, F. Dettori, B. Dey, A. Di Canto, H. Dijkstra, F. Dordei, M. Dorigo, A. Dosil Suárez, A. Dovbnya, K. Dreimanis, L. Dufour, G. Dujany, K. Dungs, P. Durante, R. Dzhelyadin, A. Dziurda, A. Dzyuba, N. Déléage, S. Easo, M. Ebert, U. Egede, V. Egorychev, S. Eidelman, S. Eisenhardt, U. Eitschberger, R. Ekelhof, L. Eklund, Ch. Elsasser, S. Ely, S. Esen, H. M. Evans, T. Evans, A. Falabella, N. Farley, S. Farry, R. Fay, D. Fazzini, D. Ferguson, A. Fernandez Prieto, F. Ferrari, F. Ferreira Rodrigues, M. Ferro-Luzzi, S. Filippov, R. A. Fini, M. Fiore, M. Fiorini, M. Firlej, C. Fitzpatrick, T. Fiutowski, F. Fleuret, K. Fohl, M. Fontana, F. Fontanelli, D. C. Forshaw, R. Forty, V. Franco Lima, M. Frank, C. Frei, J. Fu, E. Furfaro, C. Färber, A. Gallas Torreira, D. Galli, S. Gallorini, S. Gambetta, M. Gandelman, P. Gandini, Y. Gao, L. M. Garcia Martin, J. García Pardiñas, J. Garra Tico, L. Garrido, P. J. Garsed, D. Gascon, C. Gaspar, L. Gavardi, G. Gazzoni, D. Gerick, E. Gersabeck, M. Gersabeck, T. Gershon, Ph. Ghez, S. Gianì, V. Gibson, O. G. Girard, L. Giubega, K. Gizdov, V. V. Gligorov, D. Golubkov, A. Golutvin, A. Gomes, I. V. Gorelov, C. Gotti, M. Grabalosa Gándara, R. Graciani Diaz, L. A. Granado Cardoso, E. Graugés, E. Graverini, G. Graziani, A. Grecu, P. Griffith, L. Grillo, B. R. Gruberg Cazon, O. Grünberg, E. Gushchin, Yu. Guz, T. Gys, C. Göbel, T. Hadavizadeh, C. Hadjivasiliou, G. Haefeli, C. Haen, S. C. Haines, S. Hall, B. Hamilton, X. Han, S. Hansmann-Menzemer, N. Harnew, S. T. Harnew, J. Harrison, M. Hatch, J. He, T. Head, A. Heister, K. Hennessy, P. Henrard, L. Henry, J. A. Hernando Morata, E. van Herwijnen, M. Heß, A. Hicheur, D. Hill, C. Hombach, P. H. Hopchev, W. Hulsbergen, T. Humair, M. Hushchyn, N. Hussain, D. Hutchcroft, M. Idzik, P. Ilten, R. Jacobsson, A. Jaeger, J. Jalocha, E. Jans, A. Jawahery, F. Jiang, M. John, D. Johnson, C. R. Jones, C. Joram, B. Jost, N. Jurik, S. Kandybei, W. Kanso, M. Karacson, J. M. Kariuki, S. Karodia, M. Kecke, M. Kelsey, I. R. Kenyon, M. Kenzie, T. Ketel, E. Khairullin, B. Khanji, C. Khurewathanakul, T. Kirn, S. Klaver, K. Klimaszewski, S. Koliiev, M. Kolpin, I. Komarov, R. F. Koopman, P. Koppenburg, A. Kosmyntseva, M. Kozeiha, L. Kravchuk, K. Kreplin, M. Kreps, P. Krokovny, F. Kruse, W. Krzemien, W. Kucewicz, M. Kucharczyk, V. Kudryavtsev, A. K. Kuonen, K. Kurek, T. Kvaratskheliya, D. Lacarrere, G. Lafferty, A. Lai, D. Lambert, G. Lanfranchi, C. Langenbruch, T. Latham, C. Lazzeroni, R. Le Gac, J. van Leerdam, J.-P. Lees, A. Leflat, J. Lefrançois, R. Lefèvre, F. Lemaitre, E. Lemos Cid, O. Leroy, T. Lesiak, B. Leverington, Y. Li, T. Likhomanenko, R. Lindner, C. Linn, F. Lionetto, B. Liu, X. Liu, D. Loh, I. Longstaff, J. H. Lopes, D. Lucchesi, M. Lucio Martinez, H. Luo, A. Lupato, E. Luppi, O. Lupton, A. Lusiani, X. Lyu, F. Machefert, F. Maciuc, O. Maev, K. Maguire, S. Malde, A. Malinin, T. Maltsev, G. Manca, G. Mancinelli, P. Manning, J. Maratas, J. F. Marchand, U. Marconi, C. Marin Benito, P. Marino, J. Marks, G. Martellotti, M. Martin, M. Martinelli, D. Martinez Santos, F. Martinez Vidal, D. Martins Tostes, L. M. Massacrier, A. Massafferri, R. Matev, A. Mathad, Z. Mathe, C. Matteuzzi, A. Mauri, B. Maurin, A. Mazurov, M. McCann, J. McCarthy, A. McNab, R. McNulty, B. Meadows, F. Meier, M. Meissner, D. Melnychuk, M. Merk, A. Merli, E. Michielin, D. A. Milanes, M.-N. Minard, D. S. Mitzel, A. Mogini, J. Molina Rodriguez, I. A. Monroy, S. Monteil, M. Morandin, P. Morawski, A. Mordà, M. J. Morello, J. Moron, A. B. Morris, R. Mountain, F. Muheim, M. Mulder, M. Mussini, D. Müller, J. Müller, K. Müller, V. Müller, P. Naik, T. Nakada, R. Nandakumar, A. Nandi, I. Nasteva, M. Needham, N. Neri, S. Neubert, N. Neufeld, M. Neuner, A. D. Nguyen, T. D. Nguyen, C. Nguyen-Mau, S. Nieswand, R. Niet, N. Nikitin, T. Nikodem, A. Novoselov, D. P. O’Hanlon, A. Oblakowska-Mucha, V. Obraztsov, S. Ogilvy, R. Oldeman, C. J. G. Onderwater, J. M. Otalora Goicochea, A. Otto, P. Owen, A. Oyanguren, P. R. Pais, A. Palano, F. Palombo, M. Palutan, J. Panman, A. Papanestis, M. Pappagallo, L. L. Pappalardo, W. Parker, C. Parkes, G. Passaleva, A. Pastore, G. D. Patel, M. Patel, C. Patrignani, A. Pearce, A. Pellegrino, G. Penso, M. Pepe Altarelli, S. Perazzini, P. Perret, L. Pescatore, K. Petridis, A. Petrolini, A. Petrov, M. Petruzzo, E. Picatoste Olloqui, B. Pietrzyk, M. Pikies, D. Pinci, A. Pistone, A. Piucci, S. Playfer, M. Plo Casasus, T. Poikela, F. Polci, A. Poluektov, I. Polyakov, E. Polycarpo, G. J. Pomery, A. Popov, D. Popov, B. Popovici, S. Poslavskii, C. Potterat, E. Price, J. D. Price, J. Prisciandaro, A. Pritchard, C. Prouve, V. Pugatch, A. Puig Navarro, G. Punzi, W. Qian, R. Quagliani, B. Rachwal, J. H. Rademacker, M. Rama, M. Ramos Pernas, M. S. Rangel, I. Raniuk, F. Ratnikov, G. Raven, F. Redi, S. Reichert, A. C. dos Reis, C. Remon Alepuz, V. Renaudin, S. Ricciardi, S. Richards, M. Rihl, K. Rinnert, V. Rives Molina, P. Robbe, A. B. Rodrigues, E. Rodrigues, J. A. Rodriguez Lopez, P. Rodriguez Perez, A. Rogozhnikov, S. Roiser, A. Rollings, V. Romanovskiy, A. Romero Vidal, J. W. Ronayne, M. Rotondo, M. S. Rudolph, T. Ruf, P. Ruiz Valls, J. J. Saborido Silva, E. Sadykhov, N. Sagidova, B. Saitta, V. Salustino Guimaraes, C. Sanchez Mayordomo, B. Sanmartin Sedes, R. Santacesaria, C. Santamarina Rios, M. Santimaria, E. Santovetti, A. Sarti, C. Satriano, A. Satta, D. M. Saunders, D. Savrina, S. Schael, M. Schellenberg, M. Schiller, H. Schindler, M. Schlupp, M. Schmelling, T. Schmelzer, B. Schmidt, O. Schneider, A. Schopper, K. Schubert, M. Schubiger, M.-H. Schune, R. Schwemmer, B. Sciascia, A. Sciubba, A. Semennikov, A. Sergi, N. Serra, J. Serrano, L. Sestini, P. Seyfert, M. Shapkin, I. Shapoval, Y. Shcheglov, T. Shears, L. Shekhtman, V. Shevchenko, A. Shires, B. G. Siddi, R. Silva Coutinho, L. Silva de Oliveira, G. Simi, S. Simone, M. Sirendi, N. Skidmore, T. Skwarnicki, E. Smith, I. T. Smith, J. Smith, M. Smith, H. Snoek, M. D. Sokoloff, F. J. P. Soler, B. Souza De Paula, B. Spaan, P. Spradlin, S. Sridharan, F. Stagni, M. Stahl, S. Stahl, P. Stefko, S. Stefkova, O. Steinkamp, S. Stemmle, O. Stenyakin, S. Stevenson, S. Stoica, S. Stone, B. Storaci, S. Stracka, M. Straticiuc, U. Straumann, L. Sun, W. Sutcliffe, K. Swientek, V. Syropoulos, M. Szczekowski, T. Szumlak, S. T’Jampens, A. Tayduganov, T. Tekampe, M. Teklishyn, G. Tellarini, F. Teubert, E. Thomas, J. van Tilburg, M. J. Tilley, V. Tisserand, M. Tobin, S. Tolk, L. Tomassetti, D. Tonelli, S. Topp-Joergensen, F. Toriello, E. Tournefier, S. Tourneur, K. Trabelsi, M. Traill, M. T. Tran, M. Tresch, A. Trisovic, A. Tsaregorodtsev, P. Tsopelas, A. Tully, N. Tuning, A. Ukleja, A. Ustyuzhanin, U. Uwer, C. Vacca, V. Vagnoni, A. Valassi, S. Valat, G. Valenti, A. Vallier, R. Vazquez Gomez, P. Vazquez Regueiro, S. Vecchi, M. van Veghel, J. J. Velthuis, M. Veltri, G. Veneziano, A. Venkateswaran, M. Vernet, M. Vesterinen, B. Viaud, D. Vieira, M. Vieites Diaz, X. Vilasis-Cardona, V. Volkov, A. Vollhardt, B. Voneki, A. Vorobyev, V. Vorobyev, C. Voß, J. A. de Vries, C. Vázquez Sierra, R. Waldi, C. Wallace, R. Wallace, J. Walsh, J. Wang, D. R. Ward, H. M. Wark, N. K. Watson, D. Websdale, A. Weiden, M. Whitehead, J. Wicht, G. Wilkinson, M. Wilkinson, M. Williams, M. P. Williams, M. Williams, T. Williams, F. F. Wilson, J. Wimberley, J. Wishahi, W. Wislicki, M. Witek, G. Wormser, S. A. Wotton, K. Wraight, K. Wyllie, Y. Xie, Z. Xu, Z. Yang, H. Yin, J. Yu, X. Yuan, O. Yushchenko, K. A. Zarebski, M. Zavertyaev, L. Zhang, Y. Zhang, A. Zhelezov, Y. Zheng, A. Zhokhov, X. Zhu, V. Zhukov, S. Zucchelli, LHCb Collaboration

**Affiliations:** 1 Centro Brasileiro de Pesquisas Físicas (CBPF), Rio de Janeiro, Brazil; 2 Universidade Federal do Rio de Janeiro (UFRJ), Rio de Janeiro, Brazil; 3 Center for High Energy Physics, Tsinghua University, Beijing, China; 4 LAPP, Université Savoie Mont-Blanc, CNRS/IN2P3, Annecy-Le-Vieux, France; 5 Clermont Université, Université Blaise Pascal, CNRS/IN2P3, LPC, Clermont-Ferrand, France; 6 CPPM, Aix-Marseille Université, CNRS/IN2P3, Marseille, France; 7 LAL, Université Paris-Sud, CNRS/IN2P3, Orsay, France; 8 LPNHE, Université Pierre et Marie Curie, Université Paris Diderot, CNRS/IN2P3, Paris, France; 9 I. Physikalisches Institut, RWTH Aachen University, Aachen, Germany; 10 Fakultät Physik, Technische Universität Dortmund, Dortmund, Germany; 11 Max-Planck-Institut für Kernphysik (MPIK), Heidelberg, Germany; 12 Physikalisches Institut, Ruprecht-Karls-Universität Heidelberg, Heidelberg, Germany; 13 School of Physics, University College Dublin, Dublin, Ireland; 14 Sezione INFN di Bari, Bari, Italy; 15 Sezione INFN di Bologna, Bologna, Italy; 16 Sezione INFN di Cagliari, Cagliari, Italy; 17 Sezione INFN di Ferrara, Ferrara, Italy; 18 Sezione INFN di Firenze, Firence, Italy; 19 Laboratori Nazionali dell’INFN di Frascati, Frascati, Italy; 20 Sezione INFN di Genova, Genoa, Italy; 21 Sezione INFN di Milano-Bicocca, Milano, Italy; 22 Sezione INFN di Milano, Milano, Italy; 23 Sezione INFN di Padova, Padua, Italy; 24 Sezione INFN di Pisa, Pisa, Italy; 25 Sezione INFN di Roma Tor Vergata, Rome, Italy; 26 Sezione INFN di Roma La Sapienza, Rome, Italy; 27 Henryk Niewodniczanski Institute of Nuclear Physics Polish Academy of Sciences, Kraków, Poland; 28 Faculty of Physics and Applied Computer Science, AGH-University of Science and Technology, Kraków, Poland; 29 National Center for Nuclear Research (NCBJ), Warsaw, Poland; 30 Horia Hulubei National Institute of Physics and Nuclear Engineering, Bucharest-Magurele, Romania; 31 Petersburg Nuclear Physics Institute (PNPI), Gatchina, Russia; 32 Institute of Theoretical and Experimental Physics (ITEP), Moscow, Russia; 33 Institute of Nuclear Physics, Moscow State University (SINP MSU), Moscow, Russia; 34 Institute for Nuclear Research of the Russian Academy of Sciences (INR RAN), Moscow, Russia; 35 Yandex School of Data Analysis, Moscow, Russia; 36 Budker Institute of Nuclear Physics (SB RAS), Novosibirsk, Russia; 37 Institute for High Energy Physics (IHEP), Protvino, Russia; 38 ICCUB, Universitat de Barcelona, Barcelona, Spain; 39 Universidad de Santiago de Compostela, Santiago de Compostela, Spain; 40 European Organization for Nuclear Research (CERN), Geneva, Switzerland; 41 Institute of Physics, Ecole Polytechnique Fédérale de Lausanne (EPFL), Lausanne, Switzerland; 42 Physik-Institut, Universität Zürich, Zurich, Switzerland; 43 Nikhef National Institute for Subatomic Physics, Amsterdam, The Netherlands; 44 Nikhef National Institute for Subatomic Physics, VU University Amsterdam, Amsterdam, The Netherlands; 45 NSC Kharkiv Institute of Physics and Technology (NSC KIPT), Kharkiv, Ukraine; 46 Institute for Nuclear Research of the National Academy of Sciences (KINR), Kiev, Ukraine; 47 University of Birmingham, Birmingham, UK; 48 H.H. Wills Physics Laboratory, University of Bristol, Bristol, UK; 49 Cavendish Laboratory, University of Cambridge, Cambridge, UK; 50 Department of Physics, University of Warwick, Coventry, UK; 51 STFC Rutherford Appleton Laboratory, Didcot, UK; 52 School of Physics and Astronomy, University of Edinburgh, Edinburgh, UK; 53 School of Physics and Astronomy, University of Glasgow, Glasgow, UK; 54 Oliver Lodge Laboratory, University of Liverpool, Liverpool, UK; 55 Imperial College London, London, UK; 56 School of Physics and Astronomy, University of Manchester, Manchester, UK; 57 Department of Physics, University of Oxford, Oxford, UK; 58 Massachusetts Institute of Technology, Cambridge, MA USA; 59 University of Cincinnati, Cincinnati, OH USA; 60 University of Maryland, College Park, MD USA; 61 Syracuse University, Syracuse, NY USA; 62 Pontifícia Universidade Católica do Rio de Janeiro (PUC-Rio), Rio de Janeiro, Brazil; 63 University of Chinese Academy of Sciences, Beijing, China; 64 Institute of Particle Physics, Central China Normal University, Wuhan, Hubei China; 65 Departamento de Fisica, Universidad Nacional de Colombia, Bogotá, Colombia; 66 Institut für Physik, Universität Rostock, Rostock, Germany; 67 National Research Centre Kurchatov Institute, Moscow, Russia; 68 Instituto de Fisica Corpuscular, Centro Mixto Universidad de Valencia-CSIC, Valencia, Spain; 69 Van Swinderen Institute, University of Groningen, Groningen, The Netherlands; 70 CERN, 1211 Geneva 23, Switzerland

## Abstract

Two new algorithms for use in the analysis of $pp$ collision are developed to identify the flavour of ${\mathrm {B}} ^0$ mesons at production using pions and protons from the hadronization process. The algorithms are optimized and calibrated on data, using ${{\mathrm {B}} ^0} \!\rightarrow D^{-}\pi ^{+}$ decays from $pp$ collision data collected by LHCb at centre-of-mass energies of 7 and 8 TeV . The tagging power of the new pion algorithm is 60% greater than the previously available one; the algorithm using protons to identify the flavour of a ${\mathrm {B}} ^0$ meson is the first of its kind.

## Introduction

Violation of $C\!P$ symmetry in the $\mathrm {B}$ system was observed for the first time in the interference between mixing and decay processes [1]. Any measurement of a decay-time-dependent asymmetry requires the determination of the flavour of the $\mathrm {B}$ meson at production. For $\mathrm {B}$ mesons produced in $pp$ collisions, this information is obtained by means of several flavour-tagging algorithms that exploit the correlations between $\mathrm {B}$ flavour and other particles in the event.

Algorithms determining the flavour content of $\mathrm {B}$ meson by using particles associated to its production are called same-side (SS) taggers. As an example, in the production of ${\mathrm {B}} ^0$ mesons from excited charged $\mathrm {B}$ mesons decaying via strong interaction to ${{\mathrm {B}} ^0} \pi ^+$, the pion charge identifies the initial flavour of the ${\mathrm {B}} ^0$ meson.^1^ A charge correlation can also arise from the hadronization process of the $\mathrm {b}$ quark. When a $\overline{{\mathrm {b}}}$ and a $\mathrm {d}$ quark hadronize as a ${\mathrm {B}} ^0$ meson, it is likely that the corresponding $\overline{{\mathrm {d}}}$ quark ends up in a charged pion ($\mathrm {u}$ $\overline{{\mathrm {d}}}$), or in an antiproton (${\overline{{\mathrm {u}}}} {\overline{{\mathrm {u}}}} {\overline{{\mathrm {d}}}}$). The ${\mathrm {B}} ^0$ meson and the pion or antiproton are produced in nearby regions of phase space. Other algorithms used at LHCb, called opposite-side (OS) taggers [2, 3], attempt to identify the flavour of the other $\mathrm {b}$ hadron produced in the same event.

A simple cut-based SS algorithm selecting pions was successfully used by LHCb for tagging ${{\mathrm {B}} ^0} \!\rightarrow {{\mathrm {J} /\uppsi }} {{\mathrm {K}} ^0_\mathrm{\scriptscriptstyle S}}$ decays [4] in the measurement of $\sin 2\beta$, and an SS kaon tagger [5] based on a neural network was used to determine the flavour of ${\mathrm {B}} ^0_{\mathrm {s}}$ mesons in measurements of the $C\!P$-violating phase $\phi _{{\mathrm {s}}}$  [6–8]. This paper presents two new SS algorithms exploiting the charge correlation of pions and protons with ${\mathrm {B}} ^0$ mesons, denoted $\mathrm SS$ $\uppi$ and $\mathrm SS$ $\mathrm {p}$. This is the first time that protons are used for flavour tagging. The two algorithms are combined into a single tagger, $\mathrm SScomb$. Both algorithms are based on multivariate selections and are optimized, calibrated and validated using ${{\mathrm {B}} ^0} \!\rightarrow D^{-}\pi ^{+}$ and ${{\mathrm {B}} ^0} \!\rightarrow {{\mathrm {K}} ^+} {{\uppi } ^-}$ decays collected by LHCb in Run 1.

The performance of a flavour-tagging algorithm is measured by its tagging efficiency $\varepsilon _{\mathrm {tag}}$, mistag fraction $\omega$, dilution *D*, and tagging power $\varepsilon _{\mathrm {eff}}$, defined as

1 $$\begin{aligned} \varepsilon _{\mathrm {tag}}= & {} \frac{R+W}{R+W+U},\quad \omega = \frac{W}{R+W},\nonumber \\ D= & {} 1-2\omega ,\quad \varepsilon _{\mathrm {eff}} = \varepsilon _{\mathrm {tag}} D^2, \end{aligned}$$

where *R*, *W*, and *U* are the numbers of correctly-tagged, incorrectly-tagged, and untagged ${\mathrm {B}} ^0$ signal candidates. The tagging power determines the sensitivity to the measurement of a decay-time-dependent $C\!P$ asymmetry [9], as it quantifies the effective reduction in the sample size of flavour-tagged ${\mathrm {B}} ^0$ candidates. It is the figure of merit used to optimize the algorithms. Each algorithm provides a decision on the flavour of the ${\mathrm {B}} ^0$ candidate and an estimate of the probability $\eta$ that this decision is incorrect. The probability is used to determine a weight applied to the ${\mathrm {B}} ^0$ candidate, in order to maximize the tagging power of a sample of ${\mathrm {B}} ^0$ mesons in a time-dependent analysis. The probabilities provided by the two SS taggers are used to combine their decisions into the $\mathrm SScomb$ decision, which can be further combined with the decision of other taggers [2, 3].

**Table 1:** Expected correlation between the flavour of a $\mathrm {B}$ meson and the hadronization products

$\mathrm {B}$ meson	Pion	Proton	Kaon
${\mathrm {B}} ^0$	${\uppi } ^+$	$\overline{{\mathrm {p}}}$	${\overline{\mathrm {K}}{}} {}^0$
${{\mathrm {B}} ^{+}}$	${\uppi } ^-$	$\overline{{\mathrm {p}}}$	${\mathrm {K}} ^-$

The expected relationship between the flavour of charged and neutral $\mathrm {B}$ mesons and the charge of the tagging particle is reported in Table 1. For a ${\mathrm {B}} ^{+}$ meson the same correlation as for a ${\mathrm {B}} ^0$ meson holds in the case of protons, but with opposite charge in the case of pions. In addition, the tagging kaons carry the same charge as pions, while they are neutral for a ${\mathrm {B}} ^0$. Since misidentified hadrons affect the tagging efficiency and the mistag fraction of charged and neutral mesons in different ways, ${\mathrm {B}} ^{+}$ decays cannot be reliably used for the tuning and calibration of the SS taggers. As a consequence, ${\mathrm {B}} ^0$ decays are used, and a time-dependent analysis is required to determine the mistag fraction.

## Detector

The LHCb detector [10, 11] is a single-arm forward spectrometer covering the pseudorapidity range $2<\eta <5$, designed for the study of particles containing $\mathrm {b}$ or $\mathrm {c}$ quarks. The detector includes a high-precision tracking system consisting of a silicon-strip vertex detector surrounding the *pp* interaction region, a large-area silicon-strip detector located upstream of a dipole magnet with a bending power of about $4\mathrm{\,Tm}$, and three stations of silicon-strip detectors and straw drift tubes placed downstream of the magnet. Regular reversal of the magnet polarity allows a quantitative assessment of detector-induced charge asymmetries. The tracking system provides a measurement of momentum, $p$, of charged particles with a relative uncertainty that varies from 0.5% at low momentum to 1.0% at 200${\mathrm {\,GeV/}c}$. The minimum distance of a track to a primary vertex (PV), the impact parameter (IP), is measured with a resolution of $(15+29/p_\mathrm{T}){\,\upmu \mathrm{m}}$, where $p_\mathrm{T}$ is the component of the momentum transverse to the beam, in ${\mathrm {\,GeV/}c}$.

Particularly relevant for this analysis is the identification of the different species of charged hadrons, which mainly relies on the information of two ring-imaging Cherenkov detectors. The first one covers the low and intermediate momentum region 2–40${\mathrm {\,GeV/}c}$ over the full spectrometer angular acceptance of 25–300$\mathrm \,mrad$. The second Cherenkov detector covers the high momentum region 15–100${\mathrm {\,GeV/}c}$ over the angular range 15–120$\mathrm \,mrad$  [12].

Photons, electrons and hadrons are identified by a calorimeter system consisting of scintillating-pad and preshower detectors, an electromagnetic calorimeter and a hadronic calorimeter. Muons are identified by a system composed of alternating layers of iron and multiwire proportional chambers. The online event selection is performed by a trigger [13], which consists of a hardware stage, based on information from the calorimeter and muon systems, followed by a software stage, which applies a full event reconstruction. At the hardware trigger stage, events are required to have a muon with high $p_\mathrm{T}$ or a hadron, photon or electron with high transverse energy in the calorimeters. The software trigger requires a two-, three- or four-track secondary vertex detached from the PV. A multivariate algorithm [14] is used for the identification of secondary vertices consistent with the decay of a $\mathrm {b}$ hadron.

Samples of simulated events are used to model the signal mass and decay-time distributions. In the simulation, *pp* collisions are generated using Pythia  [15, 16] with a specific LHCb configuration [17]. Decays of hadronic particles are described by EvtGen  [18], in which final-state radiation is generated using Photos  [19]. The interaction of the generated particles with the detector, and its response, are implemented using the Geant4 toolkit [20, 21] as described in Ref. [22].

## Development of the same-side taggers

The $\mathrm SS$ $\uppi$ and $\mathrm SS$ $\mathrm {p}$ algorithms are developed following similar strategies. A sample of ${\mathrm {B}} ^0$ mesons decaying into the flavour-specific final state $D^-\pi ^+$, with ${\mathrm {D}} ^-$ candidates reconstructed in the final state ${\mathrm {K}} ^+$ ${\uppi } ^-$ ${\uppi } ^-$, is selected using requirements similar to those presented in Ref. [23]. The sample is collected from $pp$ collisions at $\sqrt{s} =8\,\mathrm{TeV}\,$, corresponding to an integrated luminosity of 2$\text{ fb }^{-1}$. Tagging pion or proton candidates, with their charge correlated with the ${\mathrm {B}} ^0$ flavour, are selected by means of a set of loose selection requirements and a multivariate classifier, as described below. The ${{\mathrm {B}} ^0} \!\rightarrow D^{-}\pi ^{+}$ candidates are separated randomly into three disjoint subsamples of equal size. The first sample is used for training the multivariate classifiers, the second is used for determining the probability of an incorrect tagging decision, and the third is used to evaluate the calibration of the mistag probability.

The correctness of a tagging decision is evaluated by comparing the charge of the tagging particle with the ${\mathrm {B}} ^0$ decay flavour as determined by the reconstructed final state. Those ${\mathrm {B}} ^0$ candidates that have oscillated before decaying enter the training process with an incorrectly assigned production flavour. In the training phase the dilution is reduced by requiring the decay time of the reconstructed ${\mathrm {B}} ^0$ mesons to be smaller than 2.2$\mathrm{\,ps}$. This value was optimized with simulated events and reduces the fraction of oscillated candidates to about 11%, keeping 66% of the original sample.

**Fig. 1:**
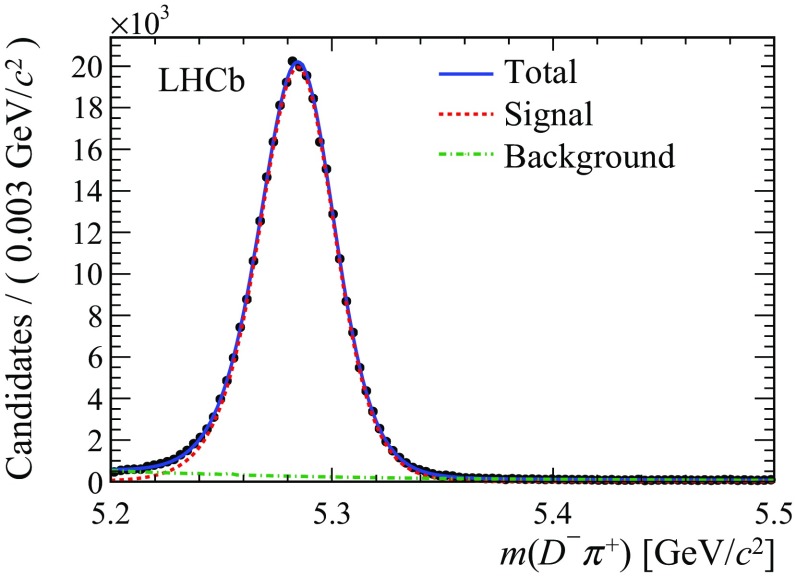
Mass distribution of ${{\mathrm {B}} ^0} \!\rightarrow D^{-}\pi ^{+}$ candidates with fit projections overlaid. Data points (*black dots*) correspond to the ${\mathrm {B}} ^0$ candidates selected in the 2 $\text{ fb }^{-1}$ data sample collected at $\sqrt{s} =8 \,\mathrm{TeV}\,$. The *solid blue curve* represents the total fit function which is the sum of signal (*red dashed*) and combinatorial background (*green dash-dotted*)

The signal and background components of the ${\mathrm {B}} ^0$ sample are determined by an unbinned maximum likelihood fit to the ${\mathrm {D}} ^-$ ${\uppi } ^+$ mass distribution of the selected candidates in the region [5.2, 5.5]${\mathrm {\,GeV\!/}c^2}$. The signal is described by a Johnson’s $S_U$ distribution [24], while the combinatorial background is modelled by the sum of an exponential function and a constant. All parameters are free to vary in the fit. A small component of ${{\mathrm {B}} ^0} \!\rightarrow D^-K^+$ decays ($\sim$1.2% as estimated from simulation), with the kaon misidentified as a pion, is neglected in the fit. The number of signal candidates in the full 2$\text{ fb }^{-1}$ sample, estimated by the mass fit and shown in Fig. 1, is 300 370 ± 674. The fit to the mass distribution is used to assign event-by-event weights (sWeights), using the *sPlot* technique [25]. The weights are subsequently used to subtract the background contribution when training the $\mathrm SS$ $\uppi$ and $\mathrm SS$ $\mathrm {p}$ classifiers and in the fits to the ${\mathrm {B}} ^0$ decay-time distribution.

The loose selection requirements reduce the multiplicity of pion (proton) candidates to 2.3 (1.7) per ${{\mathrm {B}} ^0} \!\rightarrow D^{-}\pi ^{+}$ signal candidate, and are reported in Table 2. Only tracks with hits in all tracking detectors are considered as tagging candidates. The following observables are used: the $\chi ^2/\mathrm {ndf}$ of the track fit, where ndf is the number of degrees of freedom, the track transverse momentum $p_{\mathrm {T}}^{\mathrm {track}}$, the ratio between the track impact parameter with respect to the PV associated to the ${\mathrm {B}} ^0$ meson and the error on this variable $\mathrm {IP}/\sigma _{\mathrm {IP}}$, the ratio between the track impact parameter with respect to any other PV in the event and its error $\mathrm {IP}_{\mathrm {PU}}/\sigma _{\mathrm {IP}_{\mathrm {PU}}}$, the difference between the logarithms of the likelihood of the proton and pion hypothesis $\log L_p -\log L_{\pi }$, or kaon and pion hypothesis $\log L_K -\log L_{\pi }$. The likelihoods for the various mass hypothesis are determined using the track and the Cherenkov angle information, as described in Ref. [26]. For particles passing the loose selection criteria the efficiency to identify a pion is 89% with a kaon misidentification probability of 2%, while the efficiency to identify a proton is 92% with a pion misidentification probability of 5%. Since mutually exclusive particle identification criteria are imposed, a given tagging track is identified either as a pion or as a proton. If more than one PV is reconstructed in the event, the PV associated to the ${\mathrm {B}} ^0$ meson is the one which has the smallest increase in the vertex-fit $\chi ^2$ when adding the ${\mathrm {B}} ^0$ meson to the PV.

Additional requirements are introduced on the system formed by the tagging particle and the reconstructed ${\mathrm {B}} ^0$ meson. They are applied to the total transverse momentum of the system $p_{\mathrm {T}}^{\mathrm {tot}}$, the difference between the pseudorapidity of the ${\mathrm {B}} ^0$ candidate and the tagging particle $\Delta \eta$, the azimuthal angle $\Delta \phi$ between the ${\mathrm {B}} ^0$ candidate and the tagging particle, and the difference between the invariant mass of the system and the mass of the ${\mathrm {B}} ^0$ and of the tagging particle $\Delta Q = m({{\mathrm {B}} ^0} +h)- m({{\mathrm {B}} ^0}) - m(h)$, where *h* denotes the hadron, $\pi$ or *p*. The vertex formed by the ${\mathrm {B}} ^0$ meson and the tagging particle is required to have the $\chi ^2$ of vertex fit $\chi ^2_{B^0-\mathrm {track}}$, less than 100.

**Table 2:** Loose selection requirements for the $\mathrm SS$ $\uppi$ and $\mathrm SS$ $\mathrm {p}$ algorithms. The variables used as input for the BDT classifiers are indicated by $\checkmark$

Variable	$\mathrm SS$ $\uppi$		$\mathrm SS$ $\mathrm {p}$	
	Selection	BDT	Selection	BDT
$\chi ^2_{\mathrm {track}}/\mathrm {ndf}$	$<\!\!3$	$\checkmark$	$<\!\!3$	–
$p_{\mathrm {T}}^{\mathrm {track}}$ [GeV/c]	$>\!\!0.4$	$\checkmark$	$>\!\!0.4$	$\checkmark$
$p^{\mathrm {track}}$ [GeV/c]	–	$\checkmark$	–	$\checkmark$
$\mathrm {IP}/\sigma _{\mathrm {IP}}$	$<\!\!4$	$\checkmark$	$<\!\!4$	$\checkmark$
$\mathrm {IP}_{\mathrm {PU}}/\sigma _{\mathrm {IP}_{\mathrm {PU}}}$	$>\!\!3$	–	–	–
$\log L_p -\log L_{\pi }$	$<\!\!5$	–	$>\!\!5$	$\checkmark$
$\log L_K -\log L_{\pi }$	$<\!\!5$	$\checkmark$	–	–
$p_{\mathrm {T}}^{{{\mathrm {B}} ^0}}$ [GeV/c]	–	$\checkmark$	–	–
$p_{\mathrm {T}}^{\mathrm {tot}}$ [GeV/c]	$>\!\!3$	$\checkmark$	$>\!\!3$	$\checkmark$
$\chi ^2_{B^0-\mathrm {track}}$	$<\!\!100$	–	$<\!\!100$	–
$\Delta Q$ [GeV/c$^2$]	$<\!\!1.2$	$\checkmark$	$<\!\!1.3$	$\checkmark$
$\Delta \eta$	$<\!\!1.2$	$\checkmark$	$<\!\!1.2$	$\checkmark$
$\Delta \phi$ [rad]	$<\!\!1.1$	$\checkmark$	$<\!\!1.2$	–
$\Delta R$	–	$\checkmark$	–	$\checkmark$
$PV_{\mathrm {tracks}}$	–	$\checkmark$	–	$\checkmark$

**Fig. 2:**
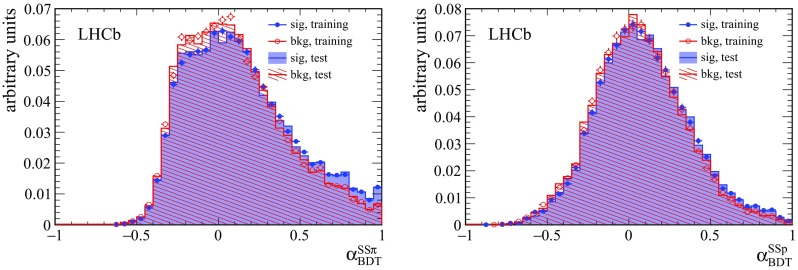
Distribution of the BDT output of signal (correct-tag decision) and background (wrong-tag decision) tagging particles, for (*left*) $\mathrm SS$ $\uppi$ and (*right*) $\mathrm SS$ $\mathrm {p}$ taggers. In case of multiple tagging candidates per ${\mathrm {B}} ^0$ candidate, only the candidate with the highest BDT output value is shown

**Fig. 3:**
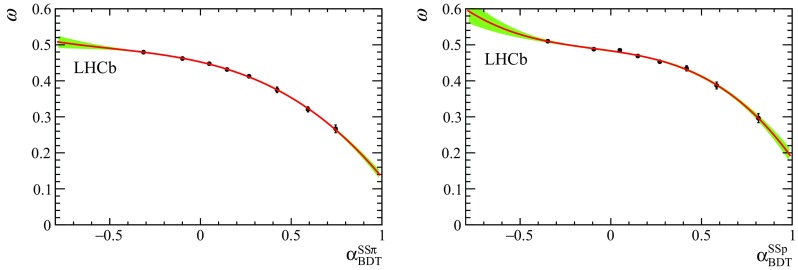
Measured average mistag fraction in bins of (*left*) $\mathrm SS$ $\uppi$ and (*right*) $\mathrm SS$ $\mathrm {p}$ BDT output. The plots are obtained with the test sample of background-subtracted ${{\mathrm {B}} ^0} \!\rightarrow D^{-}\pi ^{+}$ candidates. The *green-shaded area* shows the confidence range within $\pm 1\sigma$

The multivariate classifiers used for the selection of the tagging particles are boosted decision trees (BDT) [27] using the AdaBoost [28] method to enhance and to increase the stability with respect to statistical fluctuations. This choice has been shown to be optimal with respect to the achievable tagging power. The classifiers take most of the above observables as input, as specified in Table 2. In addition the BDTs use the following variables: the momentum of the tagging particle $p^{\mathrm {track}}$, the transverse momentum of the ${\mathrm {B}} ^0$ candidate $p_{\mathrm {T}}^{{{\mathrm {B}} ^0}}$, the separation of tagging particle and the ${\mathrm {B}} ^0$ candidate $\Delta R = \sqrt{\Delta \phi ^2 + \Delta \eta ^2}$, and the number of tracks contributing to the PV fit $PV_{\mathrm {tracks}}$. The sWeights are used to subtract the contribution of background ${\mathrm {B}} ^0$ candidates in the training of the classifiers. The charge of the tagging particle determines the flavour of the ${\mathrm {B}} ^0$ candidate. In case of multiple tagging particle candidates per ${\mathrm {B}} ^0$ candidate, the tagging particle with the highest BDT output value is chosen. The BDT outputs, $\alpha _{\mathrm {BDT}}$, are shown in Fig. 2. The global separation between signal and background is small, but enough to provide useful information to determine the flavour of the ${\mathrm {B}} ^0$ candidate, as shown below.

**Table 3:** Calibration parameters for the $\mathrm SS$ $\uppi$, $\mathrm SS$ $\mathrm {p}$ and $\mathrm SScomb$ taggers where the first uncertainties are statistical and the second are systematic

	$\mathrm SS$ $\uppi$	$\mathrm SS$ $\mathrm {p}$	$\mathrm SScomb$
$\langle \eta \rangle$	0.444	0.461	0.439
$\overline{p}_0$	$0.446 \pm 0.003 \pm 0.001$	$0.468 \pm 0.004 \pm 0.001$	$0.441 \pm 0.003 \pm 0.002$
$\overline{p}_1$	$1.05 \pm 0.05 \pm 0.01$	$1.04 \pm 0.08 \pm 0.02$	$0.99 \pm 0.04 \pm 0.02$
$\Delta p_0$	$-0.0028 \pm 0.0036 \pm 0.0016$	$-0.0218 \pm 0.0048 \pm 0.0016$	$-0.0056 \pm 0.0036 \pm 0.0018$
$\Delta p_1$	$0.015 \pm 0.074 \pm 0.014$	$0.140 \pm 0.112 \pm 0.019$	$0.052 \pm 0.060 \pm 0.017$
$A_{\mathrm {tag}}$	$-0.001 \pm 0.007 \pm 0.007$	$0.008 \pm 0.009 \pm 0.007$	$0.002 \pm 0.007 \pm 0.007$

## Evaluation and calibration of mistag probability

### The SS$\pi$ and SSp taggers

The BDT output is transformed into an estimate of the mistag probability through linear regression. The decay-time distribution of all tagged ${\mathrm {B}} ^0$ candidates is considered and the dilution due to mixing is decoupled by means of a full time-dependent analysis. Tagged ${\mathrm {B}} ^0$ candidates are divided into eight bins of the BDT output and for each bin the probability of an incorrect tagging decision is determined from an unbinned maximum likelihood fit to the distribution of the measured decay time *t* of the candidates, using the sWeights. The probability density function (PDF) for the signal is described as

2 $$\begin{aligned} \mathcal{S}(t,q)= & {} \mathcal{N}~a(t)~e^{-t'/\tau _d} ( 1 + q(1-2\omega ) \cos (\Delta m_d~t'))\nonumber \\&\otimes \mathcal{R}(t-t'), \end{aligned}$$

where $t'$ represents the true decay time, $\mathcal N$ is a normalization factor, $\omega$ is the average mistag fraction in the bin, *q* is the mixing state ($q=+1$ when the flavour at production and the flavour at decay are the same, $q=-1$ otherwise), $\mathcal{R}(t-t')$ is the decay-time resolution and *a*(*t*) is the decay-time acceptance. The ${\mathrm {B}} ^0$ lifetime $\tau _d$, and the mixing frequency $\Delta m_d$, are fixed in the fit to their known values [29].

Equation 2 is obtained under the assumption of zero width difference $\Delta \Gamma _d$ and neglecting the production and detection asymmetries between ${\mathrm {B}} ^0$ and ${\overline{\mathrm {B}}{}} {}^0$. The decay-time resolution is modelled by a Gaussian function with a fixed width of 50 $\mathrm \,fs$, as determined from simulation. The decay-time acceptance *a*(*t*), is described by a parametric function based on cubic splines [30] whose nodes have fixed position and whose parameters are determined from data. Figure 3 shows the measured average mistag rate per subsample, interpolated with a third-order polynomial that represents $\eta$ as a function of $\alpha _{\mathrm {BDT}}$, for the $\mathrm SS$ $\uppi$ and $\mathrm SS$ $\mathrm {p}$ taggers.

This polynomial parametrization is then used to determine the mistag probability $\eta (\alpha _{\mathrm {BDT}})$ of a ${\mathrm {B}} ^0$ candidate. Tagging particles with $\eta (\alpha _{\mathrm {BDT}})>0.5$ are rejected. With the third subsample of ${\mathrm {B}} ^0$ candidates, it is checked that the estimated mistag probability corresponds to the true value by measuring the mistag fraction $\omega$ with an unbinned likelihood fit to the decay-time distribution of the ${\mathrm {B}} ^0$ candidates. Possible differences between the mistag probability of ${\mathrm {B}} ^0$ and ${\overline{\mathrm {B}}{}} {}^0$ mesons may arise from the different interaction cross-sections of hadrons and antihadrons in the detector material and from differences in detection efficiencies of positive and negative hadrons. They are taken into account in the decay-time fit by defining the variables

3 $$\begin{aligned} \overline{\omega } = (\omega ^{{{\mathrm {B}} ^0}} + \omega ^{{{\overline{\mathrm {B}}{}} {}^0}})/2,\quad \Delta \omega = \omega ^{{{\mathrm {B}} ^0}} - \omega ^{{{\overline{\mathrm {B}}{}} {}^0}}, \end{aligned}$$

where $\omega ^{{{\mathrm {B}} ^0}}$ and $\omega ^{{{\overline{\mathrm {B}}{}} {}^0}}$ are the mistag fractions related to ${\mathrm {B}} ^0$ and ${\overline{\mathrm {B}}{}} {}^0$. Assuming a linear relation between the measured and estimated mistag fractions, the calibration functions are written as

4 $$\begin{aligned} \begin{matrix} \omega ^{{{\mathrm {B}} ^0}}(\eta ) = p_0^{{{\mathrm {B}} ^0}} + p_1^{{{\mathrm {B}} ^0}}(\eta - \langle \eta \rangle ), \\ \omega ^{{{\overline{\mathrm {B}}{}} {}^0}}(\eta ) = p_0^{{{\overline{\mathrm {B}}{}} {}^0}} + p_1^{{{\overline{\mathrm {B}}{}} {}^0}}(\eta - \langle \eta \rangle ), \end{matrix} \end{aligned}$$

where $p_i^{{{\mathrm {B}} ^0}}$ and $p_i^{{{\overline{\mathrm {B}}{}} {}^0}}$ (with $i=0,1$) are the calibration parameters. The average calibration parameters and the differences between the ${\mathrm {B}} ^0$ and ${\overline{\mathrm {B}}{}} {}^0$ parameters are defined as

5 $$\begin{aligned} \overline{p}_i = (p_i^{{{\mathrm {B}} ^0}} + p_i^{{{\overline{\mathrm {B}}{}} {}^0}})/2,\quad \Delta p_i = p_i^{{{\mathrm {B}} ^0}} - p_i^{{{\overline{\mathrm {B}}{}} {}^0}}. \end{aligned}$$

The use of the arithmetic mean $\langle \eta \rangle$ of the $\eta$ distribution aims at decorrelating $p_0$ and $p_1$. A perfect calibration corresponds to $\overline{p}_0=\langle \eta \rangle$ and $\overline{p}_1=1$.

A difference in the number of reconstructed and tagged ${\mathrm {B}} ^0$ and ${\overline{\mathrm {B}}{}} {}^0$ mesons arises from several possible sources. Two of these sources are considered in the fit by introducing an asymmetry in the detection efficiency of the final state particles, defined as

6 $$\begin{aligned} A_{\mathrm {det}} = \frac{\varepsilon _{\mathrm {det}}^{D^+\pi ^-}-\varepsilon _{\mathrm {det}}^{D^-\pi ^+}}{\varepsilon _{\mathrm {det}}^{D^+\pi ^-}+\varepsilon _{\mathrm {det}}^{D^-\pi ^+}}, \end{aligned}$$

and an asymmetry of the tagging efficiencies, defined as

7 $$\begin{aligned} A_{\mathrm {tag}} = \frac{ \varepsilon _{\mathrm {tag}}^{{{\overline{\mathrm {B}}{}} {}^0}} - \varepsilon _{\mathrm {tag}}^{{{\mathrm {B}} ^0}} }{ \varepsilon _{\mathrm {tag}}^{{{\overline{\mathrm {B}}{}} {}^0}} +\varepsilon _{\mathrm {tag}}^{{{\mathrm {B}} ^0}} }. \end{aligned}$$

With these additional inputs, the PDF becomes

8 $$\begin{aligned} \mathcal{S}(t,q)= & {} \mathcal{N}~a(t)~e^{-t'/\tau _d} (C_{\mathrm {cosh}} + C_{\mathrm {cos}} \cos (\Delta m_d~t'))\nonumber \\&\otimes \mathcal{R}(t-t'). \end{aligned}$$

The coefficients $C_{\mathrm {cosh}}$ and $C_{\mathrm {cos}}$ are

9 $$\begin{aligned} C_{\mathrm {cosh}}=&(1-r~A_{\mathrm {det}})\Bigl (1-\frac{a_{\mathrm {sl}}^d}{2}~\frac{1+r}{2}\Bigr )\nonumber \\&\times \Biggl ((1+A_{\mathrm {prod}}+A_{\mathrm {tag}})\Bigl (\frac{1-d}{2}+d(\omega +\Delta \omega )\Bigr ) \nonumber \\&+ (1-A_{\mathrm {prod}}-A_{\mathrm {tag}})\Bigl (\frac{1+d}{2}-d(\omega -\Delta \omega )\Bigr )\Bigl (1+\frac{a_{sl}^d}{2}\Bigr )\Biggr ), \nonumber \\ C_{\mathrm {cos}}=&-r(1-r~A_{\mathrm {det}})\Bigl (1-\frac{a_{\mathrm {sl}}^d}{2}~\frac{1+r}{2}\Bigr )\nonumber \\&\times \Biggl ((1+A_{\mathrm {prod}}+A_{\mathrm {tag}})\Bigl (\frac{1-d}{2}+d(\omega +\Delta \omega )\Bigr )\nonumber \\&- (1-A_{\mathrm {prod}}-A_{\mathrm {tag}})\Bigl (\frac{1+d}{2}-d(\omega -\Delta \omega )\Bigr )\Bigl (1+\frac{a_{\mathrm {sl}}^d}{2}\Bigr )\Biggr ), \end{aligned}$$

where *r* is the $\mathrm {B}$ meson flavour at decay ($r=+1$ for ${{\mathrm {B}} ^0} \rightarrow D^- \pi ^+$, $r=-1$ for ${{\overline{\mathrm {B}}{}} {}^0} \rightarrow D^+ \pi ^-$) and *d* is the tagging decision ($d=+1$ for $\pi ^+$ ($\overline{p}$), $d=-1$ for $\pi ^-$ (*p*)). These coefficients also take into account the production asymmetry, $A_{\mathrm {prod}} = \frac{N_{{{\overline{\mathrm {B}}{}} {}^0}}-N_{{{\mathrm {B}} ^0}}}{N_{{{\overline{\mathrm {B}}{}} {}^0}}+N_{{{\mathrm {B}} ^0}}}$, and the asymmetry in mixing, or flavour-specific asymmetry, $a_{\mathrm {sl}}^{d}$. These two asymmetries cannot be distinguished from the tagging and detection asymmetries and are fixed in the fit. The production asymmetry is fixed to the value measured in Ref. [31], $A_{\mathrm {prod}}=(-0.58 \pm 0.70)\%$, while $a_{\mathrm {sl}}^{d}$ is fixed to the world average $a_{\mathrm {sl}}^d = (-0.15 \pm 0.17)\%$ [32]. The effect of their uncertainties on the calibration parameters is included in the systematic uncertainty.

The calibration parameters for the two taggers obtained in the fit to the calibration sample of ${{\mathrm {B}} ^0} \!\rightarrow D^{-}\pi ^{+}$ decays are reported in Table 3. The correlations between the calibration parameters are below 10%, except for the asymmetry of the tagging efficiencies, which has a correlation of about 16% with $\Delta p_0$ and $\Delta p_1$ and about 64% with $A_{\mathrm {det}}$. For the $\mathrm SS$ $\uppi$ tagger, $A_{\mathrm {tag}}$, $\Delta p_0$ and $\Delta p_1$ are zero within one standard deviation, showing no significant difference in tagging behaviour between ${\mathrm {B}} ^0$ and ${\overline{\mathrm {B}}{}} {}^0$ decays. For the $\mathrm SS$ $\mathrm {p}$ tagger, it is found that $\Delta p_0<0$, as a consequence of the higher interaction cross-section of anti-protons with matter compared to protons. A similar effect is reported for kaon taggers [5]. The fit result of the detection asymmetry is comparable for the two taggers ($A_{\mathrm {det}}^\mathrm{SS{\uppi }} =(-0.87 \pm 0.48)\%$, $A_{\mathrm {det}}^{{\mathrm {SS}}{ p }}= (-0.66 \pm 0.62)\%$) and in agreement with that found in Ref. [33]. The systematic uncertainties on the parameters will be described in Sect. 5.

After calibration, the total tagging power of the sample is calculated as

10 $$\begin{aligned} \varepsilon _{\mathrm {eff}} = \frac{\sum _{i=1}^{N_{\mathrm {tag}}}(1-2{\overline{\omega }}(\eta _i))^2s_i}{\sum _{j=1}^{N} s_j} \end{aligned}$$

where $s_i$ is the sWeight of the candidate *i*, *N* and $N_{\mathrm {tag}}$ are the numbers of total and tagged candidates, having mistag probability $\eta _i$, and the average mistag fraction ${\overline{\omega }}(\eta _i)$ is calculated using Eqs. 3 and 4. Candidates with a mistag probability larger than 0.5 are considered untagged and are removed from the sum in the numerator, effectively setting $\omega (\eta _i) = 0.5$. The tagging performances for the $\mathrm SS$ $\uppi$ and $\mathrm SS$ $\mathrm {p}$ taggers are reported in Table 4.

**Table 4:** Tagging efficiencies and tagging power of the $\mathrm SS$ $\uppi$, $\mathrm SS$ $\mathrm {p}$ and $\mathrm SScomb$ algorithms. The $\mathrm SScomb$ efficiencies are shown splitting the sample in candidates tagged exclusively by $\mathrm SS$ $\uppi$ or $\mathrm SS$ $\mathrm {p}$, or by both. As explained in the text, there is a large overlap between the $\mathrm SS$ $\uppi$ and $\mathrm SS$ $\mathrm {p}$ taggers

Tagger	Sample	$\varepsilon _{\mathrm {tag}}$ [$\%$]	$\varepsilon _{\mathrm {eff}}$ [$\%$]
$\mathrm SS$ $\uppi$		$71.96 \pm 0.23$	$1.69 \pm 0.10$
$\mathrm SS$ $\mathrm {p}$		$38.56 \pm 0.15$	$0.53 \pm 0.05$
$\mathrm SScomb$	$\mathrm SS$ $\uppi$ only	$35.91 \pm 0.14$	$0.95 \pm 0.08$
	$\mathrm SS$ $\mathrm {p}$ only	$8.75 \pm 0.10$	$0.12 \pm 0.02$
	$\mathrm SS$ $\uppi$ and $\mathrm SS$ $\mathrm {p}$	$34.74 \pm 0.15$	$1.04 \pm 0.07$
	total	$79.40 \pm 0.23$	$2.11 \pm 0.11$

**Fig. 4:**
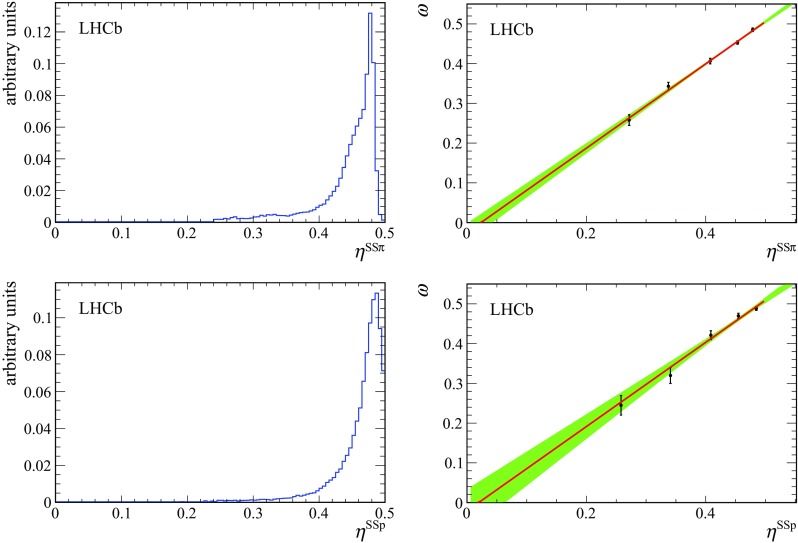
(*Top left*) distribution of the mistag probability $\eta ^\mathrm{SS{\uppi }}$ and (*top right*) measured mistag fraction $\omega$ as a function of $\eta ^\mathrm{SS{\uppi }}$. (*Bottom left*) distribution of the mistag probability $\eta ^\mathrm{SS{\mathrm {p}}}$ and (*bottom right*) measured mistag fraction $\omega$ as a function of $\eta ^\mathrm{SS{\mathrm {p}}}$. The *green-shaded area* shows the 68% confidence range

**Fig. 5:**
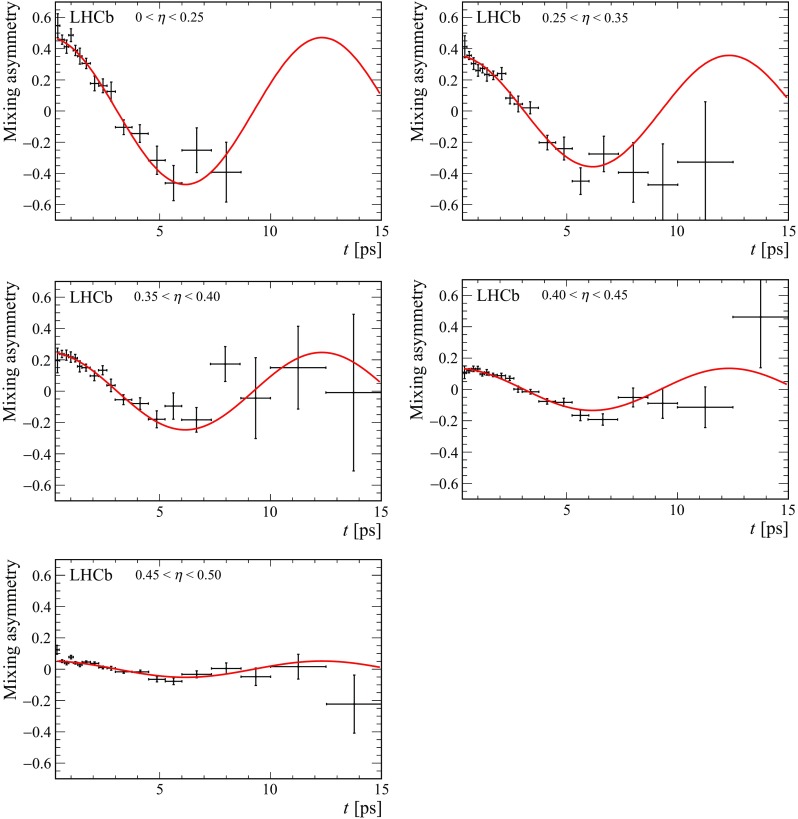
Mixing asymmetry in bins of mistag probability using the $\mathrm SS$ $\uppi$ tagger

**Fig. 6:**
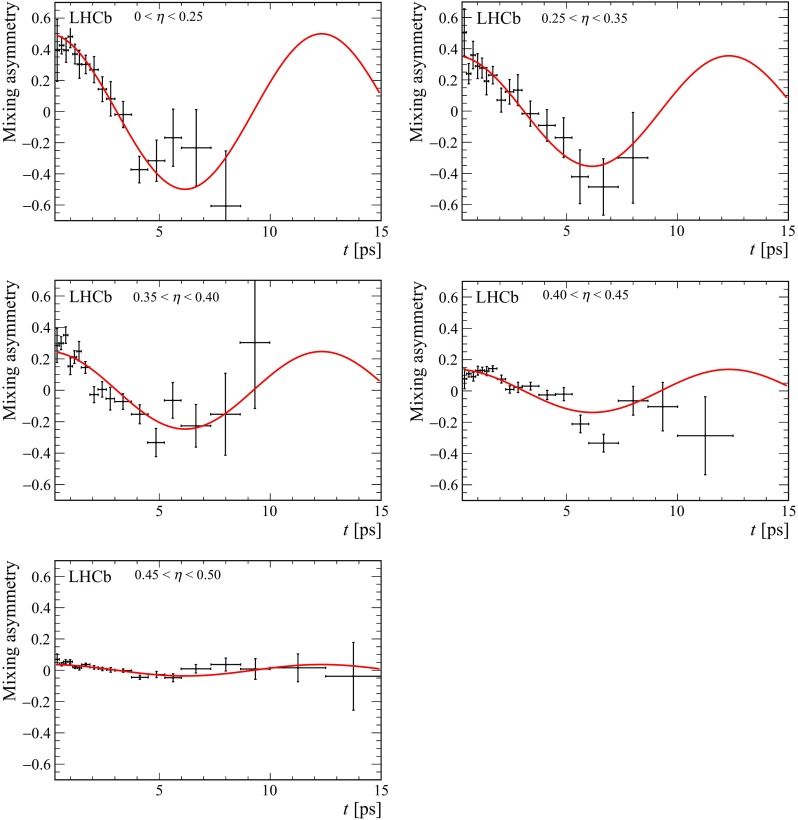
Mixing asymmetry in bins of mistag probability using the $\mathrm SS$ $\mathrm {p}$ tagger

The fit of the decay-time distribution is repeated after dividing events into bins of predicted mistag probability. The distribution of $\eta$ and the dependence of the measured mistag fraction on $\eta$ are shown in Fig. 4 with the linear fits superimposed, demonstrating the expected linearity. In Figs. 5 and 6 the time-dependent mixing asymmetries $A=\frac{N^{\mathrm {unmix}}-N^{\mathrm {mix}}}{N^{\mathrm {unmix}}+N^{\mathrm {mix}}}$ are shown for each of the five bins.

### The SScomb tagger

Even though a given tagging particle can be selected by only one of the $\mathrm SS$ $\uppi$ or the $\mathrm SS$ $\mathrm {p}$ taggers, both taggers may find a candidate track in the same event. About 50% of the candidates tagged by $\mathrm SS$ $\uppi$ are also tagged by $\mathrm SS$ $\mathrm {p}$, and 80% of the candidates tagged by $\mathrm SS$ $\mathrm {p}$ are also tagged by $\mathrm SS$ $\uppi$. When both taggers provide a decision, they are combined into a single decision. Since the correlation between the $\mathrm SS$ $\uppi$ and $\mathrm SS$ $\mathrm {p}$ decisions, and between their mistag probabilities, is found to be small, it is neglected when combining them using the following formulae

11 $$\begin{aligned} p(b)= & {} \prod _i \bigg (\frac{1+d_i}{2} - d_i(1-\eta _i) \bigg ),\nonumber \\ p(\overline{b})= & {} \prod _i \bigg (\frac{1-d_i}{2}+d_i(1-\eta _i)\bigg ), \end{aligned}$$

where p($\mathrm {b}$) and p($\overline{{\mathrm {b}}}$) are the probabilities that the signal $\mathrm {B}$ meson contains a $\mathrm {b}$ or a $\overline{{\mathrm {b}}}$ quark respectively, and $d_i$ is the tagging decision of the tagger $i=$ $\mathrm SS$ $\uppi$, $\mathrm SS$ $\mathrm {p}$. The normalized probabilities are

12 $$\begin{aligned} P(\overline{b}) = \frac{p(\overline{b})}{p(\overline{b}) + p(b)}, \quad P(b) = 1 - P(\overline{b}). \end{aligned}$$

For $P(\overline{b}) > P(b)$ the combined tagging decision is $d=+1$ and the final mistag probability is $\eta = P(b)$. Otherwise, the combined tagging decision and the mistag probability are $d=-1$ and $\eta =P(\overline{b})$.

The combination procedure, which assumes no correlation, is validated by checking the combined mistag probability a posteriori. Assuming a linear relation between the predicted mistag probability and the true mistag fraction, the calibration parameters in the overlapping sample give $(\overline{p}_0 - \langle \eta \rangle )= 0.010 \pm 0.005$ and $(\overline{p}_1-1)= 0.01 \pm 0.08$. The calibration is repeated on the sample of all ${\mathrm {B}} ^0$ candidates tagged by the $\mathrm SScomb$ tagger, and the calibration parameters derived from the unbinned likelihood fit with the PDF of Eq. 8, reported in Table 3, demonstrate its validity. The performance of $\mathrm SScomb$ is reported in Table 4. The total tagging power obtained by the combined algorithm is $(2.11\pm 0.11)\%$, a relative increase of 25% compared to that provided by the $\mathrm SS$ $\uppi$ tagger alone.

A higher tagging power can be obtained from the combination of the SScomb tagger with the OS tagger. The OS tagger is the combination of various OS tagging algorithms using electrons and muons from semileptonic decays of $\mathrm {b}$ hadrons, kaons from $b \rightarrow c \rightarrow s$ decay chains and the inclusive reconstruction of a secondary vertex of the decay products of the opposite side $\mathrm {b}$ hadron. The SS and OS taggers are found to be uncorrelated, so their combination follows the same procedure as the combination of $\mathrm SS$ $\uppi$ and $\mathrm SS$ $\mathrm {p}$ into $\mathrm SScomb$. The calibration of the combined mistag probability is verified a posteriori with a fit of the decay-time distribution of the ${\mathrm {B}} ^0$ candidates. For ${{\mathrm {B}} ^0} \!\rightarrow D^{-}\pi ^{+}$ decays, the total tagging efficiency and the total tagging power are $(84.48 \pm 0.26)\%$ and $(5.14 \pm 0.15)\%$, respectively. On the same sample, the use of the OS tagger only provides a tagging efficiency and a tagging power of $(37.95 \pm 0.15)\%$ and $(3.52 \pm 0.17)\%$, respectively.

## Validation and systematic uncertainties

A possible dependence of the calibration parameters of the SS taggers on properties of the event sample is checked by repeating the calibration after splitting the data according to the data-taking conditions (magnet polarity), global event properties (total number of reconstructed tracks, number of primary vertices) or according to the kinematic properties of the ${\mathrm {B}} ^0$ meson (transverse momentum, pseudorapidity and azimuthal angle). The average mistag probability has a weak dependence on the number of tracks in the event. On the other hand, it decreases as a function of the transverse momentum since the number of random tracks decreases at high $p_T^B$. The tagging efficiency is nearly constant for pions, while the requirement on proton identification reduces the number of proton candidates at high $p_T^B$. A similar dependence is present versus the pseudorapidity of the ${\mathrm {B}} ^0$ meson. Since the average mistag fraction and the $p_0$ parameter decrease with increasing $p_\mathrm{T}^{{\mathrm {B}} ^0}$, the calibration remains valid in all subsamples, with variations below two standard deviations.

The portability of the mistag calibration, from the training data sample to other data samples and other ${\mathrm {B}} ^0$ decay modes, is validated using an independent sample of ${{\mathrm {B}} ^0} \!\rightarrow D^{-}\pi ^{+}$ decays collected at $\sqrt{s}$ = 7 TeV (corresponding to an integrated luminosity of 1$\text{ fb }^{-1}$) and a sample of ${{\mathrm {B}} ^0} \!\rightarrow {{\mathrm {K}} ^+} {{\uppi } ^-}$ decays collected at $\sqrt{s}$ = 8 TeV (corresponding to an integrated luminosity of 2$\text{ fb }^{-1}$). The same selection criteria and fitting procedure as described above are used for the ${{\mathrm {B}} ^0} \!\rightarrow D^{-}\pi ^{+}$ validation sample at $\sqrt{s}$ = 7 TeV . The calibration parameters for the $\mathrm SS$ $\uppi$, $\mathrm SS$ $\mathrm {p}$, and $\mathrm SScomb$ taggers determined from an unbinned maximum likelihood fit to the decay-time distribution are compatible with those derived in the 8 TeV sample. Consistent values of tagging power are found for all taggers.

The selection criteria and the mass model for the ${{\mathrm {B}} ^0} \!\rightarrow {{\mathrm {K}} ^+} {{\uppi } ^-}$ candidates are described in Ref. [34]. The decay-time acceptance is parametrized using cubic splines with six nodes, whose positions are fixed and whose coefficients are free in the fit. The decay-time resolution is described by a Gaussian function with parameters determined from simulation. The parameters shown in Table 5 demonstrate a good portability of the mistag calibration, with $\overline{p}_0 - \langle \eta \rangle \approx 0$ and $\overline{p}_1 - 1 \approx 0$ as expected. A lower tagging power is measured in this channel, giving $(1.06 \pm 0.09 )$%, $(0.42 \pm 0.06 )$%, and $(1.37 \pm 0.13 )$% for $\mathrm SS$ $\uppi$, $\mathrm SS$ $\mathrm {p}$ and $\mathrm SScomb$, respectively, as expected from the lower average $p_\mathrm{T}$ of the selected ${\mathrm {B}} ^0$ candidates.

**Table 5:** Calibration parameters for the ${{\mathrm {B}} ^0} \!\rightarrow {{\mathrm {K}} ^+} {{\uppi } ^-}$ decay sample. Uncertainties are statistical only

Tagger	$\langle \eta \rangle$	$\overline{p}_0$	$\overline{p}_1$	$\Delta p_0$	$\Delta p_1$	$A_{\mathrm {tag}}$
$\mathrm SS$ $\uppi$	0.456	$0.452 \pm 0.003$	$1.06 \pm 0.09$	$0.0053 \pm 0.0042$	$0.047 \pm 0.115$	$-0.009 \pm 0.008$
$\mathrm SS$ $\mathrm {p}$	0.467	$0.459 \pm 0.004$	$0.80 \pm 0.14$	$-0.0138 \pm 0.0051$	$0.025 \pm 0.141$	$0.008 \pm 0.009$
$\mathrm SScomb$	0.452	$0.457 \pm 0.003$	$0.94 \pm 0.07$	$-0.0034 \pm 0.0040$	$0.079 \pm 0.086$	$0.007 \pm 0.007$

Several sources of systematic uncertainties on the calibration parameters are studied and the associated uncertainties are reported in Table 6. Uncertainties related to the mass model and background unfolding procedure are assessed by repeating the calibration replacing the sWeights derived in the fit to the mass distribution of all ${\mathrm {B}} ^0$ candidates by the sWeights derived after restricting the sample to tagged ${\mathrm {B}} ^0$ candidates. In a second test, the signal mass model is replaced by a Hypatia function [35] convolved with a Gaussian function. The sum in quadrature of the variations of the calibration parameters observed in the two tests is taken as uncertainty on the mass model.

Uncertainties related to the decay-time acceptance model are assessed by changing the number of nodes in the cubic splines from six to nine and are found to be negligible. A negligible uncertainty is associated to the decay-time resolution model. The mistag model uncertainties are assessed by comparing the calibration parameters derived in the nominal fit and those derived in fits with the mistag probability binned in categories. Five, seven and nine bins are tested and the largest observed variation of the parameters is taken as a systematic uncertainty. Differences between the results of the two implementations of the time-dependent fit are due to the dependence of the mistag probability on the decay time. Pseudoexperiments are generated where the mistag probability has the same dependence on time as in data and are fitted with the two approaches. The difference in parameters is similar to or smaller than that observed in data.

**Table 6:** Systematic uncertainties on the calibration parameters of $\mathrm SS$ $\uppi$, $\mathrm SS$ $\mathrm {p}$ and $\mathrm SScomb$ taggers. The total systematic uncertainty is the squared sum of all contributions. A dash indicates a value negligible with respect to the quoted precision

Tagger	Source	$\sigma (\overline{p}_0)$	$\sigma (\overline{p}_1)$	$\sigma (\Delta p_0)$	$\sigma (\Delta p_1)$	$\sigma (A_{\mathrm {tag}})$
$\mathrm SS$ $\uppi$	Mass model	–	–	–	0.001	–
	Mistag model	0.001	0.01	0.0002	0.007	–
	Decay model	0.001	0.01	0.0016	0.012	0.007
	Total	0.001	0.01	0.0016	0.014	0.007
$\mathrm SS$ $\mathrm {p}$	Mass model	–	–	0.0002	0.004	–
	Mistag model	0.001	0.02	–	0.014	0.001
	Decay model	0.001	0.01	0.0016	0.012	0.007
	Total	0.001	0.02	0.0016	0.019	0.007
$\mathrm SScomb$	Mass model	–	–	0.0008	0.005	–
	Mistag model	0.002	0.02	0.0004	0.010	0.001
	Decay model	0.001	0.01	0.0016	0.012	0.007
	Total	0.002	0.02	0.0018	0.017	0.007

**Table 7:** Systematic uncertainties related to the decay-time model. A dash indicates a value negligible with respect to the quoted precision

Source	$\sigma (\overline{p}_0)$	$\sigma (\overline{p}_1)$	$\sigma (\Delta p_0)$	$\sigma (\Delta p_1)$	$\sigma (A_{\mathrm {tag}})$
$\Delta \Gamma$	0.00013	–	–	–	0.001
$A_{\mathrm {prod}}$	0.00002	–	–	–	0.007
$a_{\mathrm {sl}}^{\mathrm {d}}$	–	–	–	–	–
$C\!P$ violation	0.00124	0.01	0.0016	0.012	0.002
Total	0.001	0.01	0.0016	0.012	0.007

Uncertainties related to neglecting $\Delta \Gamma _d$ and possible $C\!P$ violation in the ${{\mathrm {B}} ^0} \!\rightarrow D^{-}\pi ^{+}$ decays in the decay-time fit, are studied by performing pseudoexperiments in which changes associated with the parameter under study are incorporated in the generation and neglected in the subsequent fit. Terms proportional to the relevant $C\!P$ parameters are added to the PDF in Eq. 8 and the values of the parameters are taken from Ref. [32]. The associated systematic uncertainties are taken to be the changes in the calibration parameters with respect to perfect calibration ($\overline{p}_0=\langle \eta \rangle$, $\overline{p}_1$=1), used in the generation. Uncertainties related to the variation of $A_{\mathrm {prod}}$ and $a_{\mathrm {sl}}^d$, which are fixed in the decay-time fit, are evaluated with pseudoexperiments where the parameters are varied within their uncertainties. The uncertainties are determined in the $\mathrm SS$ $\uppi$ configuration and attributed to both taggers. A breakdown of the systematic uncertainties related to the decay-time model is shown in Table 7.

## Conclusion

Two new same-side algorithms are developed to determine the production flavour of ${\mathrm {B}} ^0$ mesons using pions and protons from the hadronization process. This is the first time that protons are used to identify the flavour of a ${\mathrm {B}} ^0$ meson. The algorithms are optimized and calibrated on data using ${{\mathrm {B}} ^0} \!\rightarrow D^{-}\pi ^{+}$ decays. The calibration parameters of the taggers are reported in Table 3. The efficiency and mistag probability of the taggers depend on the kinematic properties of the ${\mathrm {B}} ^0$ decay mode under study. Estimated mistag probabilities match the true mistag fraction throughout the phase space. The new $\mathrm SS$ $\uppi$ tagger provides a tagging power that is greater by 60% relative to the previous algorithm using pions, employed in Ref. [4]. Adding the combination of the two new algorithms to the existing OS taggers provides a relative increase of the total tagging power of about 40%.

## Footnotes

1 The inclusion of charge-conjugate processes is implied throughout the paper, unless otherwise noted.
